# Pudendal nerve block decreases narcotic requirements and time spent in post-anesthesia care units in patients undergoing primary inflatable penile prosthesis implantation

**DOI:** 10.1038/s41443-024-00870-1

**Published:** 2024-05-17

**Authors:** Michael Zhu, Kevin Labagnara, Justin Loloi, Mustufa Babar, Arshia Aalami Harandi, Azizou Salami, Ari Bernstein, Jonathan Davila, Meenakshi Davuluri, Charbel Chalouhy, Pedro Maria

**Affiliations:** 1 Albert Einstein College of Medicine, Bronx, NY USA; 2 Department of Urology, Montefiore Medical Center and Albert Einstein College of Medicine, Bronx, NY USA; 3 Department of Urology, New York University Langone Health, New York, NY USA

**Keywords:** Surgery, Erectile dysfunction

## Abstract

Efforts to minimize narcotic usage following inflatable penile prosthesis (IPP) implantation are vital, considering the current opioid epidemic in the United States. We aimed to determine whether pudendal nerve block (PNB) utilization in a multiethnic population undergoing primary IPP implantation can decrease rates of post-operative opiate usage. A single-institution, retrospective study was conducted on patients who underwent primary IPP implantation between December 2015 and June 2022. PNB usage and intra- and post-operative outcomes were analyzed using multivariate binary logistic regression. 449 patients were included, with 373 (83.1%) in the PNB group. Median time (minutes) spent in the post-anesthesia care unit (PACU) (1499 [119–198] vs. 235 [169–322], *p* < 0.001) was significantly lower in the PNB group. There were no significant differences in intra-operative and PACU morphine milligram equivalents or post-operative safety outcomes between groups. However, fewer patients in the PNB group called for pain medications post-operatively (10.2% vs 19.7%, *p* = 0.019). Multivariate analysis revealed a significantly decreased operative time (B −6.23; 95%CI −11.28, −1.17; *p* = 0.016) and decreased time in recovery (B: −81.62; 95%CI: −106.49, −56.76, *p* < 0.001) in the PNB group. PNB decreases post-operative opioid analgesic requirements and time spent in PACU in patients undergoing a primary IPP implantation and thus may represent an attractive, non-opioid adjunct.

## Introduction

The United States Department of Health and Human Services has recognized the opioid epidemic as a public health emergency in the United States [1], with opioid overdose being the leading cause of accidental death in 2022 [2]. Post-operative pain control with opioids is a common practice; however, efforts to minimize their usage are of critical importance given the current crisis. Additionally, regulations placed on physicians to reduce opioid prescribing has led to the development of novel strategies for non-opioid-based pain management. However, in the field of urology, the literature on non-opioid post-operative recovery is scarce [3, 4].

Inflatable penile prosthesis (IPP) represents a gold standard surgical treatment for medically-refractory erectile dysfunction [5, 6]. There are various pain-control regimens that combine different classes of analgesics for post-operative pain control following IPP implantation [7]. Achieving optimal post-operative pain control begins in the pre-operative holding area through the desensitization of pain receptors across the central and peripheral nervous systems. Pre-operative administration of acetaminophen, nonsteroidal anti-inflammatory drugs/cyclooxygenase-2 (NSAIDs/COX-2) inhibitors, and gabapentin/pregabalin has demonstrated their pivotal role in reducing post-operative narcotic usage, both in urologic and non-urologic surgeries [8]. While not studied in urology, a recent comparative analysis in dental medicine comparing meloxicam and ibuprofen revealed superior overall pain control, enhanced sustainability of pain relief, and a dose-dependent reduction in pain among patients receiving meloxicam [9].

Post-operative pain control after IPP surgery can be enhanced by targeting nerve endings and receptors in penile tissues. The dorsal penile nerve, formed by converging nerve fibers, carries signals through the pudendal nerve to the spinal cord (S2–S4), then to the thalamus and sensory cortex. Anesthesia can be applied at various points along this pathway, including the dorsal nerve, perineal nerve, pudendal nerve, and/or S2–S4 nerve roots [7]. Several studies explored intra-operative analgesia for post-operative pain control. Raynor et al., for instance, found that the dorsal penile nerve block reduced early post-operative pain but did not impact post-operative narcotic use [10]. Additionally, Xie et al. studied the effectiveness of a combination of penile dorsal nerve and ring blocks, while Hsu et al. investigated the effectiveness of a crural block. Both studies noted a decrease in early post-operative pain, although rates of post-operative narcotic use were not reported [11, 12]. Furthermore, although not extensively studied in urology, ultrasound-guided hydro dissection of peripheral nerves with an anesthetic, saline, and/or 5% dextrose in water may offer a method of reducing post-operative pain, especially in the fields of pain and musculoskeletal medicine [13–15].

Pudendal nerve block (PNB) provides regional perineal and penile anesthesia and is an attractive option to maximize pain control while minimizing pelvic surgery post-operative narcotic use. For long-term pain therapy, a combination of lidocaine, bupivacaine, and a steroid (such as dexamethasone) is most commonly used and can provide 30 days or more of relief [16]. However, there is a paucity of studies assessing whether utilization of PNBs decreases intra- and post-operative narcotic requirements following IPP surgery [17, 18]. Thus, the primary objective of this study is to determine whether PNB utilization in the immediate pre-operative setting decreases intra- and post-operative narcotic requirements in patients from a diverse, multiethnic population undergoing primary IPP surgery. Secondary objectives were to assess PNB utilization on intra- and post-operative characteristics and 30-day safety outcomes. We hypothesized that PNB usage will decrease intra- and post-operative narcotic requirements, leading to quicker recovery times.

## Materials and Methods

A single institution retrospective study was conducted of patients who underwent primary IPP surgery between December 2015 and June 2022. Institutional review board approval was obtained prior to study commencement. In our current practice, the use of pre-operative PNB prior to IPP surgery was initiated in 2016. All patients undergoing IPP surgery following initiation were given a PNB unless they already had an IPP that was getting exchanged. General anesthesia was administered to all patients. Those undergoing placement of a semi-rigid prosthesis were excluded, along with those suffering from a chronic pain condition (such as fibromyalgia) or a history of substance abuse. No sample size calculations were performed because of the expected small sample size and the inability to perform the necessary calculations due to the scarcity of previous literature evaluating intra- and post-operative narcotic requirements following IPP surgery.

**Fig. 1:**
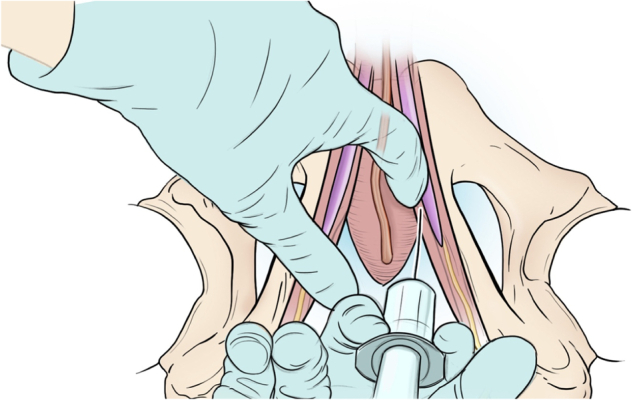
Delivery of PNB prior to IPP surgery. The urethra is moved away from the injection site, and then the needle is inserted medially to the ischial tuberosity [21].

A PNB consisted of 0.5% ropivacaine 20 ml, 1% lidocaine 20 ml, and dexamethasone 4 mg. PNBs were performed bilaterally using the transperineal approach with the patient in the lithotomy position. The needle was inserted medially to the ischial tuberosity, advanced posterolaterally until it touched the ischial spine, and then directed through the sacrospinous ligament, extending one centimeter medially from the ischial spine. Local anesthesia was administered following a negative aspiration [19, 20]. Fig. 1 illustrates the delivery of a PNB prior to IPP surgery [21]. IPPs were left inflated after surgery. All patients were admitted and discharged on the same day of the surgery. Patients were discharged with 20 tablets of acetaminophen-codeine 300–30 mg and instructed to take one tablet by mouth every four hours as needed for pain.

Electronic medical records were queried for demographic data such as age, race, preferred language, BMI, and medical comorbidities. Operative logs were accessed for intra-operative characteristics and outcomes such as IPP cylinder/reservoir size, rear tip extender (yes/no), and operative time. Total amounts of fentanyl, hydromorphone, and oxycodone administered both intra-operatively (post-induction) and in recovery were obtained from medication logs and converted to oral morphine milligram equivalents (MME) per Center for Disease Control conversion rates (https://www.cdc.gov/drugoverdose/pdf/calculating_total_daily_dose-a.pdf). Provider notes from 1-week and 1-month follow-up visits and medical staff notes documenting telephones logs were used to record 30-day safety and opiate use metrics, excluding the prescription of acetaminophen-codeine given during discharge.

Baseline characteristics of PNB (yes/no) were summarized and analyzed using a Student’s t-test for continuous variables or a Chi-square test for categorical variables. The Mann-Whitney U test was used to compare the medians of non-normally distributed outcome measures. PNB usage and calling for opiates within 30 days (yes/no), total operative time (minutes), and post-anesthesia care unit (PACU) recovery time to discharge (minutes) were analyzed using binary logistic regression and multiple linear regression. A multivariate regression model was used to adjust for the effects of age, BMI, surgical approach (penoscrotal vs infrapubic), and concurrent surgery (circumcision, penile remodeling, urethral dilation, hydrocele drainage, and/or glansplasty). Missing data was excluded from statistical analysis. All *p*-values were 2-sided with statistical significance set at *p* < 0.05. All statistical analyses were performed using SPSS version 28.0 (IBM, Armonk, NY).

## Results

A total of 617 patients were assessed for eligibility, of which 449 patients met inclusion; 168 patients were excluded for the following reasons: placement of a semi-rigid prosthesis (*n* = 59), suffering from a chronic pain condition (*n* = 81), history of substance abuse (*n* = 28). Of the 449 patients, 373 (83.1%) were in the PNB group and 76 (16.9%) in the non-PNB group (Table 1). The majority of patients were of Hispanic race (*n* = 309, 69.4%), with 304 reporting Spanish as their preferred language (68.0%). Smoking status and rates of prior prostatectomy, pelvic radiation therapy, Peyronie’s disease, diabetes, and hypertension were similar between groups (*p* > 0.05 for all groups). The infrapubic approach (49.3% vs 26.3%, *p* = 0.001) and Coloplast penile implant (97.4% vs 83.1%, *p* = 0.001) were used more often in the PNB group. Rates of any concurrent surgery (29.8% vs 13.2%, *p* = 0.003), particularly circumcisions (*p* = 0.004), were more likely to be undergone in the PNB group, although this difference was not observed for other concurrent procedures such as penile remodeling (*p* = 0.33). The distribution of implant cylinder size, reservoir size, and rate of rear tip extender usage was similarly distributed between the groups (all *p* > 0.05).

**Table 1:** Baseline characteristics of pudendal block versus non-pudendal block groups.

	All Patients	No PNB	PNB	*P*-value
*N* (%)	449	76 (16.9)	373 (83.1)	
Pre-operative				
Age, median (IQR)	63 (58–68)	63 (58–68)	63 (58–68)	0.83
BMI, median (IQR)	28.3 (25.8–31.6)	28.3 (25.8–31.6)	28.3 (25.6–31.6)	0.58
Race, *N* (%)				0.63
Non-Hispanic White	10 (2.2)	1 (1.3)	9 (2.4)	
Non-Hispanic Black	58 (13.0)	10 (13.2)	48 (13.0)	
Hispanic	309 (69.4)	50 (65.8)	259 (70.2)	
Other	68 (15.3)	15 (19.7)	53 (14.4)	
Preferred Language, *N* (%)				0.73
English	140 (31.3)	24 (31.6)	116 (31.3)	
Spanish	304 (68.0)	52 (68.4)	252 (67.9)	
Other	3 (0.7)	0 (0.0)	3 (0.8)	
Etiology/Comorbidities, *N* (%)				
Prostatectomy	114 (25.4)	22 (28.9)	92 (24.7)	0.43
XRT	32 (7.1)	3 (3.9)	29 (7.8)	0.24
Peyronie’s Disease	28 (6.2)	4 (5.3)	24 (6.4)	0.70
Diabetes	256 (57.0)	46 (60.5)	210 (56.3)	0.50
Hypertension	326 (72.6)	57 (75.0)	269 (72.1)	0.61
Smoker				0.41
Never	287 (63.9)	45 (59.2)	242 (64.9)	
Former	126 (28.1)	26 (34.2)	100 (26.8)	
Active	36 (8.0)	5 (6.6)	31 (8.3)	
Intra-operative				
Approach, *N* (%)				0.001
Penoscrotal	245 (54.6)	56 (73.7)	189 (50.7)	
Infrapubic	204 (45.4)	20 (26.3)	184 (49.3)	
Concurrent Surgery, *N* (%)				
Any	121 (26.9)	10 (13.2)	111 (29.8)	0.003
Circumcision	89 (19.8)	6 (7.9)	83 (22.3)	0.004
Penile Remodeling	36 (8.0)	4 (5.3)	32 (8.6)	0.33
Other	4 (0.9)	0 (0.0)	4 (1.1)	0.36
Implant Cylinder Size (cm), *N* (%)				0.11
< 18	46 (10.3)	8 (10.7)	38 (10.2)	
18–19	163 (36.4)	34 (45.3)	129 (34.6)	
20–21	165 (36.8)	27 (36.0)	138 (37.0)	
22+	74 (16.5)	6 (8.0)	68 (18.2)	
Reservoir Size ≥ 100cc	163 (36.7)	30 (42.3)	133 (35.7)	0.29
Rear Tip Extender, *N* (%)	229 (51.2)	41 (55.4)	188 (50.4)	0.43

*PNB* Pudendal nerve block, *IQR* interquartile range, *XRT* Radiation therapy.

Characteristics were analyzed using Student’s t-test for continuous variables and Chi-square test for categorical variables. The Mann-Whitney U test was used to compare the medians of non-normally distributed outcome measures.

Although median [IQR] operative time in minutes was similar between the groups (64 [55–78] vs 67 [56–81], *p* = 0.40) (Table 2), multivariate analysis revealed a significantly decreased operative time in the PNB group (B: −6.23; 95%CI: −11.28, −1.17; *p* = 0.016) when adjusting for age, BMI, infrapubic approach, and any concurrent surgery(s) (Table 3). The infrapubic approach was independently associated with a longer operative time (B: 10.41; 95%CI: 6.81, 14.01; *p* < 0.001) along with whether a concurrent surgery occurred (B: 13.58; 95%CI: 9.66, 17.49; *p* < 0.001). A similar relationship was observed with PNB and decreased time in recovery (B: −81.62; 95%CI: −106.49, −56.76, *p* < 0.001).

**Table 2:** Intra-operative, recovery, and post-operative outcomes of pudendal block versus non-pudendal block groups.

	All Patients	No PNB	PNB	*P*-value
Intra-operative				
Operative Time (minutes), median (IQR)	65 (55–79)	67 (56–81)	64 (55–78)	0.40
Post-induction MME, median (IQR)	150 (0–300)	150 (32–300)	150 (0–300)	0.083
Recovery				
Time spent in PACU (minutes), median (IQR)	158 (122–226)	235 (169–322)	149 (119–198)	**<** 0.001
Opiates Given in PACU, *N* (%)	286 (65.6)	48 (76.2)	238 (63.8)	0.056
PACU MME, median (IQR)	15 (0-83)	15 (3–75)	15 (0–90)	0.81
Post-operative				
Drain insertion, *N* (%)	25 (5.6)	5 (6.6)	20 (5.4)	0.67
Calls for pain meds, *N* (%)	53 (11.8)	15 (19.7)	38 (10.2)	0.019
Discharged with catheter, *N* (%)	36 (8.0)	7 (9.2)	29 (7.8)	0.67
30-Day ED Presentation, *N* (%)	52 (11.6)	10 (13.2)	42 (11.3)	0.64
90-Day Infection, *N* (%)	20 (4.5)	4 (5.3)	16 (4.3)	0.71

*PNB* Pudendal nerve block, *IQR* Interquartile range, *MME* Morphine milligram equivalents, *PACU* Post-anesthesia care unit, *ED* Emergency department.

Outcome measures were analyzed using Student’s *t*-test for continuous variables and Chi-square test for categorical variables. The Mann-Whitney U test was used to compare the medians of non-normally distributed outcome measures.

**Table 3:** Univariate and multivariate analysis of pudendal block usage and on select outcome metrics.

	Univariate		Multivariate	
	Coeff (95% CI)	*P*- value	Coeff (95% CI)	*P*-value
Total Operative Time				
PNB	−2.06 (−7.46, 3.33)	0.45	−6.23 (−11.28, −1.17)	0.016
Age	−0.01 (−0.25, 0.23)	0.94	0.09 (−0.14, 0.31)	0.45
BMI	0.62 (0.21, 1.03)	0.003	0.38 (−0.01, 0.77)	0.053
Infrapubic Approach	10.08 (6.40, 13.77)	**<** 0.001	10.41 (6.81, 14.01)	**<** 0.001
Concurrent Surgery	12.87 (8.81, 16.93)	**<** 0.001	13.58 (9.66, 17.49)	**<** 0.001
Total Recovery Time				
PNB	−79.77 (−104.03, −55.50)	**<** 0.001	−81.62 (−106.49, −56.76)	**<** 0.001
Age	−0.06 (−1.20, 1.08)	0.92	0.10 (−1.00, 1.20)	0.86
BMI	−0.29 (−2.23, 1.65)	0.77	−0.45 (−2.35, 1.45)	0.64
Infrapubic Approach	−10.86 (−28.75, 7.03)	0.23	−0.77 (−18.48, 16.93)	0.93
Concurrent Surgery	5.71 (−14.24, 25.65)	0.57	12.90 (−6.37, 32.17)	0.19
	**OR (95% CI)**	***P*-value**	**OR (95% CI)**	***P*-value**
Calling for Pain Meds				
PNB	0.46 (0.24–0.89)	0.021	0.56 (0.28–1.11)	0.096
Age	0.99 (0.96–1.03)	0.57	0.99 (0.96–1.03)	0.61
BMI	0.97 (0.91–1.04)	0.39	0.98 (0.92–1.05)	0.54
Infrapubic Approach	0.52 (0.29–0.97)	0.038	0.62 (0.33–1.16)	0.14
Concurrent Surgery	0.77 (0.39–1.52)	0.45	0.86 (0.43–1.73)	0.67

*PNB* Pudendal nerve block, *OR* Odds ratio, *CI* Confidence interval, *BMI* Body mass index.

Outcome metrics were analyzed using binary logistic regression and multiple linear regression.

Intra-operative (*p* = 0.083) and PACU total MME (*p* = 0.81) were not significantly different between groups. Rates of drain insertion (5.4% vs 6.6%, *p* = 0.67) and discharge with a catheter (7.8% vs 9.2%, *p* = 0.67) were similar between groups as well. In the 30 days following discharge, compared to the no PNB group, fewer patients in the PNB group called the physician’s office asking for pain medication (10.2% vs 19.7%, *p* = 0.019), while 30-day ED presentation rates (11.3% vs 13.2%, *p* = 0.64) and 90-day infection rates (4.3% vs 5.3%, *p* = 0.71) remained similar.

## Discussion

The United States opioid epidemic remains a national public health crisis [22]. Recent studies have illustrated that patients receiving opioids at seven days after minor surgery were 44% more likely to experience long-term opioid use [23, 24]. For IPP surgery, multimodal analgesic regimens that utilize combinations of different non-opioid analgesics to control post-operative pain have been well studied and implemented [17]. PNBs may be considered a potentially viable method to provide an additional layer of pain control during IPP implantation, while also possibly reducing narcotic use both intra- and post-operatively. To our knowledge, there have only been two studies conducted that have analyzed intra- and post-operative narcotic requirements after IPP implantation with PNB in patients; both studies relied on relatively homogenous study populations [17, 18]. Our study not only represents the largest cohort of IPP patients undergoing PNB but also depicts outcomes in a patient population that is historically underrepresented in clinical research.

Given the magnitude of the opioid epidemic, efforts to minimize narcotic usage in the post-operative setting has become increasingly important, with nerve blocks becoming increasingly utilized in urologic procedures [25]. For IPP implantation, dorsal penile nerve blocks have been previously shown to provide significant reductions in pain in the immediate post-operative period, with no differences in narcotic usage between patients receiving the block and those in the placebo group [10].

Specifically, PNB has been studied extensively in several fields and procedures outside of urology and IPP implantation. A review by Mongelli et al. found demonstrable reductions in opioid consumption, post-operative pain, complications, and length of stay after PNB in patients undergoing hemorrhoidectomy [26]. Another study found that general anesthesia plus bilateral PNB decreased the incidence and severity of catheter-related bladder discomfort for the first 12 h after transurethral resection of the prostate or transurethral resection of bladder tumor [27]. More relatedly, Hecht et al. found that compared to a caudal block, PNB in patients undergoing hypospadias repair was associated with a significantly shorter post-operative length of stay, although there was no significant difference in intra- or post-operative opioid requirements [28]. However, PNB has also been associated with several common side effects, such as injection site discomfort, bleeding, and infection, as well as serious side effects including pudendal nerve damage or adjacent organ damage [29]. Furthermore, when compared to PNB for IPP surgeries, penile crural blocks has also shown promising results of being reliable, simple, and safer with fewer complications, including lower rates of postoperative pain [12].

Although pre-operative PNB decreases intra-operative opioid requirements in patients undergoing IPP implantation, there is scarce research regarding narcotic requirements and clinical outcomes following PNB for IPP surgery. As IPP surgery is becoming more common [30], it is increasingly important to explore ways to provide safe intra- and post-operative care for patients while simultaneously aiming to reduce post-operative narcotic use for pain control. Urologists have begun to explore this gap in knowledge. A recent study showed no significant difference in pain scores between patients receiving non-opioid multimodal analgesia including PNB and patients receiving traditional opioid-based patient-controlled analgesia after robot-assisted radical prostatectomy [31]. With regards to IPP surgery, we found that patients who received PNB had a significantly decreased operative time, similar to findings by Sayyid et al. [18]. The lower operative times observed in patients receiving PNB may be attributed to more effective pain control. This improved pain management could lead to a more comfortable patient, facilitating smoother progression of the surgery. Additionally, the effective pain control provided by PNBs may contribute to a more stable intra-operative environment, thereby reducing the likelihood of interruptions. Furthermore, enhanced pain management in the perineal and genital areas can optimize certain steps of the surgery, enabling surgeons to perform procedures with greater precision and speed when the patient experiences reduced discomfort.

In contrast, we found no significant difference in intra-operative narcotic requirements in patients receiving PNB versus control; however, we did find that significantly fewer patients receiving PNB were administered narcotics in the PACU compared to control. In addition, we found that patients receiving PNB were less likely to call for additional pain medications post-discharge. This result was similar to that found in a study by Lucas et al., which found that patients receiving a multimodal analgesia protocol which included intra-operative PNB for IPP implantation required fewer narcotics both during inpatient hospitalization and post-discharge [17]. Altogether, our study consisting of the largest cohort represented in the literature validates the few studies that have demonstrated reduced intra- and post-operative narcotic requirements after IPP implantation, while showing that these results hold true for a racially diverse population.

IPP surgeries are typically performed using either the penoscrotal or infrapubic surgical approach. The choice of surgical access for IPP is primarily based on the surgeon’s experience [32]. Although both approaches have high patient satisfaction rates, each has its unique strengths and challenges. The infrapubic approach offers benefits such as quicker device placement and direct visualization during reservoir insertion. Nonetheless, drawbacks may encompass challenges with pump placement, restricted corporal exposure, and an increased risk of injury to the sensory nerves of the penis. In contrast, the penoscrotal approach enhances corporal exposure and facilitates securing the pump in the dependent portion of the scrotum, with minimal risk of nerve damage [33]. To date, there is no evidence that either of these surgical approaches reduces infection rates [34]. In our study, although we observed that the infrapubic approach was associated with longer total operative times, we did not observe a relationship between a surgical approach and post-operative pain.

Limitations of our study include its retrospective nature. Anesthesiologists were not blinded to PNB status, which may introduce bias into choosing the amount of narcotics to administer in the intra- and post-operative setting. This may be better addressed with a randomized, placebo-controlled trial. Additionally, as PNBs were used only after 2016, our findings may also be influenced by the experience and learning curve of the surgeon. For example, improved surgical technique leading to decreased tissue manipulation and smaller corporotomies may be the inherent driver behind decreased pain in the PNB group, rather than the PNB itself. Furthermore, post-operative pain was not measured using standardized questionnaires and therefore cannot be verified. Additionally, there was a considerable difference in the sample size between the two groups, thereby decreasing the statistical power of the study. Moreover, our cohort was not standardized in terms of surgical approaches and concurrent surgeries as these factors may impact PNB outcomes. Finally, this study was limited to a cohort of urban patients at an academic medical center and therefore may not be generalizable to suburban or rural patient populations. However, despite the limitations, the findings of this study corroborate the clinical relevance and utility of PNB in those undergoing IPP surgery.

## Conclusions

Ultimately, in the largest cohort represented in the literature thus far, we found that PNB decreases post-operative narcotic requirements and time spent in PACU in patients undergoing a primary IPP implantation. Furthermore, we found that patients receiving PNB had a significantly decreased operative time, though we found no significant difference in intra-operative narcotic requirements between groups. Overall, PNB offers an effective, non-opioid alternative for analgesia in patients undergoing IPP – important findings given the rising utilization of IPPs and the ongoing opioid epidemic in the United States. Future research should seek to expand the generalizability of these findings and validate our observational data with randomized, blinded trials consisting of diverse populations.

## Data Availability

The datasets generated during and/or analyzed during the current study are available from the corresponding author on reasonable request.

## References

[1] Opioid Facts and Statistics. United States Department of Health and Human Services. Accessed 01/29/2024, https://www.hhs.gov/opioids/statistics/index.html

[2] Wide-ranging online data for epidemiologic research (WONDER). CDC, National Center for Health Statistics. Accessed 01/29/2024, http://wonder.cdc.gov

[3] Glare P, Aubrey KR, Myles PS. Transition from acute to chronic pain after surgery. Lancet. 2019;393:1537–46. 10.1016/s0140-6736(19)30352-6. 30983589 10.1016/S0140-6736(19)30352-6

[4] Wick EC, Grant MC, Wu CL. Postoperative Multimodal Analgesia Pain Management With Nonopioid Analgesics and Techniques: A Review. JAMA Surg. 2017;152:691–7. 10.1001/jamasurg.2017.0898. 28564673 10.1001/jamasurg.2017.0898

[5] Scott FB, Bradley WE, Timm GW. Management of erectile impotence. Use of implantable inflatable prosthesis. Urology. 1973;2:80–2. 10.1016/0090-4295(73)90224-0. 4766860 10.1016/0090-4295(73)90224-0

[6] Scott FB, Byrd GJ, Karacan I, Olsson P, Beutler LE, Attia SL. Erectile impotence treated with an implantable, inflatable prosthesis. Five years of clinical experience. Jama. 1979;241:2609–12. 439356

[7] Reinstatler L, Shee K, Gross MS. Pain Management in Penile Prosthetic Surgery: A Review of the Literature. Sex Med Rev. 2018;6:162–9. 10.1016/j.sxmr.2017.05.005. 28735683 10.1016/j.sxmr.2017.05.005

[8] Gritsenko K, Khelemsky Y, Kaye AD, Vadivelu N, Urman RD. Multimodal therapy in perioperative analgesia. Best Pract Res Clin Anaesthesiol. 2014;28:59–79. 10.1016/j.bpa.2014.03.001. 24815967 10.1016/j.bpa.2014.03.001

[9] Christensen SE, Cooper SA, Mack RJ, McCallum SW, Du W, Freyer A. A Randomized Double-Blind Controlled Trial of Intravenous Meloxicam in the Treatment of Pain Following Dental Impaction Surgery. J Clin Pharm. 2018;58:593–605. 10.1002/jcph.1058. 10.1002/jcph.1058 PMC5947566 29329493

[10] Raynor MC, Smith A, Vyas SN, Selph JP, Carson CC,3rd. Dorsal penile nerve block prior to inflatable penile prosthesis placement: a randomized, placebo-controlled trial. J Sex Med. 2012;9:2975–9. 10.1111/j.1743-6109.2012.02756.x. 22642415 10.1111/j.1743-6109.2012.02756.x

[11] Xie D, Nicholson M, Azaiza M, Gheiler V, Lopez I, Nehrenz GM. et al. Effect of operative local anesthesia on postoperative pain outcomes of inflatable penile prosthesis: prospective comparison of two medications. Int J Impot Res. 2018;30:93–6. 10.1038/s41443-018-0025-7. 29795532 10.1038/s41443-018-0025-7

[12] Hsu GL, Hsieh CH, Wen HS, Chen SC, Chen YC, Liu LJ. et al. Outpatient penile implantation with the patient under a novel method of crural block. Int J Androl. 2004;27:147–51. 10.1111/j.1365-2605.2004.00465.x. 15139969 10.1111/j.1365-2605.2004.00465.x

[13] Lam KHS, Hung CY, Chiang YP, Onishi K, Su DCJ, Clark TB. et al. Ultrasound-Guided Nerve Hydrodissection for Pain Management: Rationale, Methods, Current Literature, and Theoretical Mechanisms. J Pain Res. 2020;13:1957–68. 10.2147/jpr.S247208. 32801851 10.2147/JPR.S247208 PMC7414936

[14] Song B, Marathe A, Chi B, Jayaram P. Hydrodissection as a therapeutic and diagnostic modality in treating peroneal nerve compression. Proc Bayl Univ Med Cent. 2022;33:465–6. 10.1080/08998280.2020.1758006. 10.1080/08998280.2020.1758006 PMC7340467 32675990

[15] Wu YT, Chen SR, Li TY, Ho TY, Shen YP, Tsai CK. et al. Nerve hydrodissection for carpal tunnel syndrome: A prospective, randomized, double-blind, controlled trial. Muscle Nerve. 2019;59:174–80. 10.1002/mus.26358. 30339737 10.1002/mus.26358

[16] Antolak S Jr., Antolak C, Lendway L. Measuring the Quality of Pudendal Nerve Perineural Injections. Pain Physician. 2016;19:299–306. 27228517

[17] Lucas J, Gross M, Yafi F, DeLay K, Christianson S, El-Khatib FM. et al. A Multi-institutional Assessment of Multimodal Analgesia in Penile Implant Recipients Demonstrates Dramatic Reduction in Pain Scores and Narcotic Usage. J Sex Med. 2020;17:518–25. 10.1016/j.jsxm.2019.11.267. 31866125 10.1016/j.jsxm.2019.11.267

[18] Sayyid RK, Taylor NS, Owens-Walton J, Oberle MD, Fratino KL, Terris MK, et al. Pudendal nerve block prior to inflatable penile prosthesis implantation: decreased intra-operative narcotic requirements. Int J Impot Res. 2023;35:1–5. 10.1038/s41443-021-00495-8 34819658

[19] Das A, Soroush M, Maurer P, Hirsch I. Multicomponent penile prosthesis implantation under regional anesthesia. Tech Urol. 1999;5:92–4. 10458662

[20] Kaufman JJ. Penile prosthetic surgery under local anesthesia. J Urol. 1982;128:1190–1. 10.1016/s0022-5347(17)53416-3. 7154170 10.1016/s0022-5347(17)53416-3

[21] Lewis K, Bole R, Booher J, DeAngelo L, Fuller G, Roberts LH. et al. Anatomic transperineal pudendal nerve block before penile prosthesis placement to reduce immediate postoperative pain - description and outcomes. Urol Video J. 2023;19:100232. 10.1016/j.urolvj.2023.100232.

[22] Lyden J, Binswanger IA. The United States opioid epidemic. Semin Perinatol. 2019;43:123–31. 10.1053/j.semperi.2019.01.001. 30711195 10.1053/j.semperi.2019.01.001 PMC6578581

[23] Alam A, Gomes T, Zheng H, Mamdani MM, Juurlink DN, Bell CM. Long-term analgesic use after low-risk surgery: a retrospective cohort study. Arch Intern Med. 2012;172:425–30. 10.1001/archinternmed.2011.1827. 22412106 10.1001/archinternmed.2011.1827

[24] Brummett CM, Waljee JF, Goesling J, Moser S, Lin P, Englesbe MJ. et al. New Persistent Opioid Use After Minor and Major Surgical Procedures in US Adults. JAMA Surg. 2017;152:e170504. 10.1001/jamasurg.2017.0504. 28403427 10.1001/jamasurg.2017.0504 PMC7050825

[25] García Rojo E, García Gómez B, Manfredi C, Alonso Isa M, Medina Polo J, Carpintero Miguel M. et al. Efficacy and safety of dorsal penile nerve block before collagenase of clostridium histolyticum injections in peyronie’s disease patients: Results from a prospective pilot study. Andrologia. 2020;52:e13740. 10.1111/and.13740. 32780475 10.1111/and.13740

[26] Mongelli F, Treglia G, La Regina D, Di Giuseppe M, Galafassi J, Majno-Hurst PE. et al. Pudendal Nerve Block in Hemorrhoid Surgery: A Systematic Review and Meta-analysis. Dis Colon Rectum. 2021;64:617–31. 10.1097/dcr.0000000000001985. 33591044 10.1097/DCR.0000000000001985

[27] Xiaoqiang L, Xuerong Z, Juan L, Mathew BS, Xiaorong Y, Qin W. et al. Efficacy of pudendal nerve block for alleviation of catheter-related bladder discomfort in male patients undergoing lower urinary tract surgeries: A randomized, controlled, double-blind trial. Med (Baltim). 2017;96:e8932. 10.1097/md.0000000000008932. 10.1097/MD.0000000000008932 PMC5728874 29245259

[28] Hecht S, Piñeda J, Bayne A. Ultrasound-guided Pudendal Block Is a Viable Alternative to Caudal Block for Hypospadias Surgery: A Single-Surgeon Pilot Study. Urology. 2018;113:192–6. 10.1016/j.urology.2017.11.006. 29155191 10.1016/j.urology.2017.11.006

[29] Schmidt RA. Technique of pudendal nerve localization for block or stimulation. J Urol. 1989;142:1528–31. 10.1016/s0022-5347(17)39150-4. 2585630 10.1016/s0022-5347(17)39150-4

[30] Baas W, O’Connor B, Welliver C, Stahl PJ, Stember DS, Wilson SK. et al. Worldwide trends in penile implantation surgery: data from over 63,000 implants. Transl Androl Urol. 2020;9:31–7. 10.21037/tau.2019.09.26. 32055463 10.21037/tau.2019.09.26 PMC6995940

[31] Lee JE, Oh J, Lee JN, Ri HS, Lee CS, Yeo J. Comparison of a Non-Opioid Multimodal Analgesia Protocol with Opioid-Based Patient-Controlled Analgesia for Pain Control Following Robot-Assisted Radical Prostatectomy: A Randomized, Non-Inferiority Trial. J Pain Res. 2023;16:563–72. 10.2147/jpr.S397529. 36846203 10.2147/JPR.S397529 PMC9946841

[32] Henry GD. Historical Review of Penile Prosthesis Design and Surgical Techniques: Part 1 of a Three-Part Review Series on Penile Prosthetic Surgery. J Sex Med. 2009;6:675–81. 10.1111/j.1743-6109.2008.01145.x. 19207278 10.1111/j.1743-6109.2008.01145.x

[33] Trost LW, Boonjindasup AG, Hellstrom WJG. Comparison of infrapubic versus transcrotal approaches for inflatable penile prosthesis placement: a multi-institution report. Int J Impot Res. 2015;27:86–9. 10.1038/ijir.2014.35. 25339138 10.1038/ijir.2014.35

[34] Palmisano F, Boeri L, Cristini C, Antonini G, Spinelli MG, Franco G. et al. Comparison of Infrapubic vs Penoscrotal Approaches for 3-Piece Inflatable Penile Prosthesis Placement: Do We Have a Winner? Sex Med Rev. 2018;6:631–9. 10.1016/j.sxmr.2018.03.007. 29730314 10.1016/j.sxmr.2018.03.007

